# Structure of the Afferent Terminals in Terminal Ganglion of a Cricket and Persistent Homology

**DOI:** 10.1371/journal.pone.0037278

**Published:** 2012-05-23

**Authors:** Jacob Brown, Tomáš Gedeon

**Affiliations:** Department of Mathematical Sciences, Center for Computational Biology, Montana State University, Bozeman, Montana, United States of America; University of Salamanca-Institute for Neuroscience of Castille and Leon and Medical School, Spain

## Abstract

We use topological data analysis to investigate the three dimensional spatial structure of the locus of afferent neuron terminals in crickets *Acheta domesticus*. Each afferent neuron innervates a filiform hair positioned on a cercus: a protruding appendage at the rear of the animal. The hairs transduce air motion to the neuron signal that is used by a cricket to respond to the environment. We stratify the hairs (and the corresponding afferent terminals) into classes depending on hair length, along with position. Our analysis uncovers significant structure in the relative position of these terminal classes and suggests the functional relevance of this structure. Our method is very robust to the presence of significant experimental and developmental noise. It can be used to analyze a wide range of other point cloud data sets.

## Introduction

One of the most pressing issues in biology in general, and particularly in neuroscience, is the development of computational methods that can extract relevant information from noisy data. We are currently in a situation where there is still a lack of high precision quantitative data, but often a relative abundance of noisy and imprecise data. In the present work, we show how one can use ideas from persistent homology [1], [2], [3] to analyze the three dimensional spatial structure of the afferent neurons locus in the cercal system of a cricket. This method is applicable to any point cloud data set that exhibits an underlying topological structure corrupted by noise.

**Figure 1:**
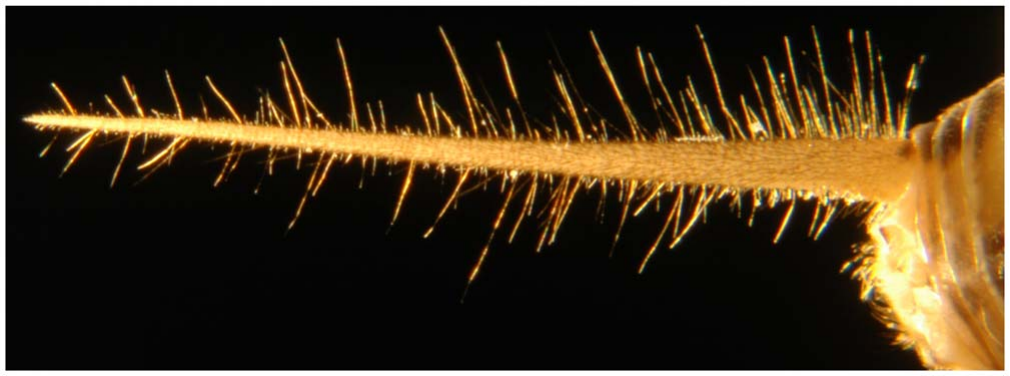
The cercus of *Acheta Domestica* with filiform hairs clearly visible. The length of the cercus is approximately 1 cm.

The cercal system in a house cricket *Acheta domesticus* (Figure 1) is a near-field flow sensor that senses fluid particle motion via an array of very thin mechanosensory hairs called filiform hairs. These hairs are distributed along two antenna-like, shallowly-tapering appendages called cerci at the rear of the abdomen. Different hairs have different directional and frequency sensitivities that are determined by the biomechanical properties of the hair and its socket. Each hair is innervated by a single neuron, whose axon projects into the terminal ganglion located near the rear end of the animal. Each axon forks into a tree-like structure. Individual branches are covered with synaptic boutons that make synaptic contact with one of the ganglion's interneurons. By staining the afferent neuron and imaging with a scanning microscope, one can locate the three dimensional coordinates of the set of synaptic boutons for a particular hair. Hairs are functionally characterized by the orientation of their socket, which determines the orientation of air motion that this hair mechanically responds to; by the length of the hair, which determines the sensitivity to frequency of air motion; and by the position on the cercus, which determines the latency of arrival of the signal to the terminal ganglion. By superimposing the sets of synaptic terminals of all hairs in all categories, we obtain the overall afferent synaptic locus. The goal of this paper is to study the topological structure of this locus and its stratification by the hair directional sensitivity and length.

### 1.1. Structure of the afferent locus

The initial experiments and analysis of the structure of the afferent locus was done in a series of papers by Jacobs and collaborators [4], [5], [6]. The first paper constructed an anatomical database consisting of twelve different identified sensory afferents for five specimens, which spanned the entire range of directional tunings seen in the system. All twelve afferents were associated with the longest mechanoreceptors (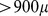
m) and, as a result of experimental accessibility, were selected from the proximal part of the cercus (i.e the initial 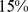 of the length from the base of the cercus). Jacobs and Theunissen [4] have shown that the locus of the afferent terminals changes continuously with the directional tuning of the corresponding hairs. In the second paper [5] the database was extended to include medium hairs (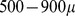
m) from the proximal part of the cercus. Their research indicates that there is no significant statistical difference between the positions of the terminals of medium hairs and those of long hairs. Finally, in an elegant paper, Jacobs and Theunissen [6] showed how the directional tuning curves of four interneurons that are downstream from the afferents arise from the overlap of the dendritic trees of the interneurons with the afferent terminal locus. In particular, the interneuron sensitivity to the motion from direction 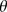 arises from the connectivity of its dendrite primarily with the terminals of afferents that innervate hairs that mechanically respond to the direction 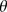.

### 1.2. Statement of the problem

**Figure 2:**
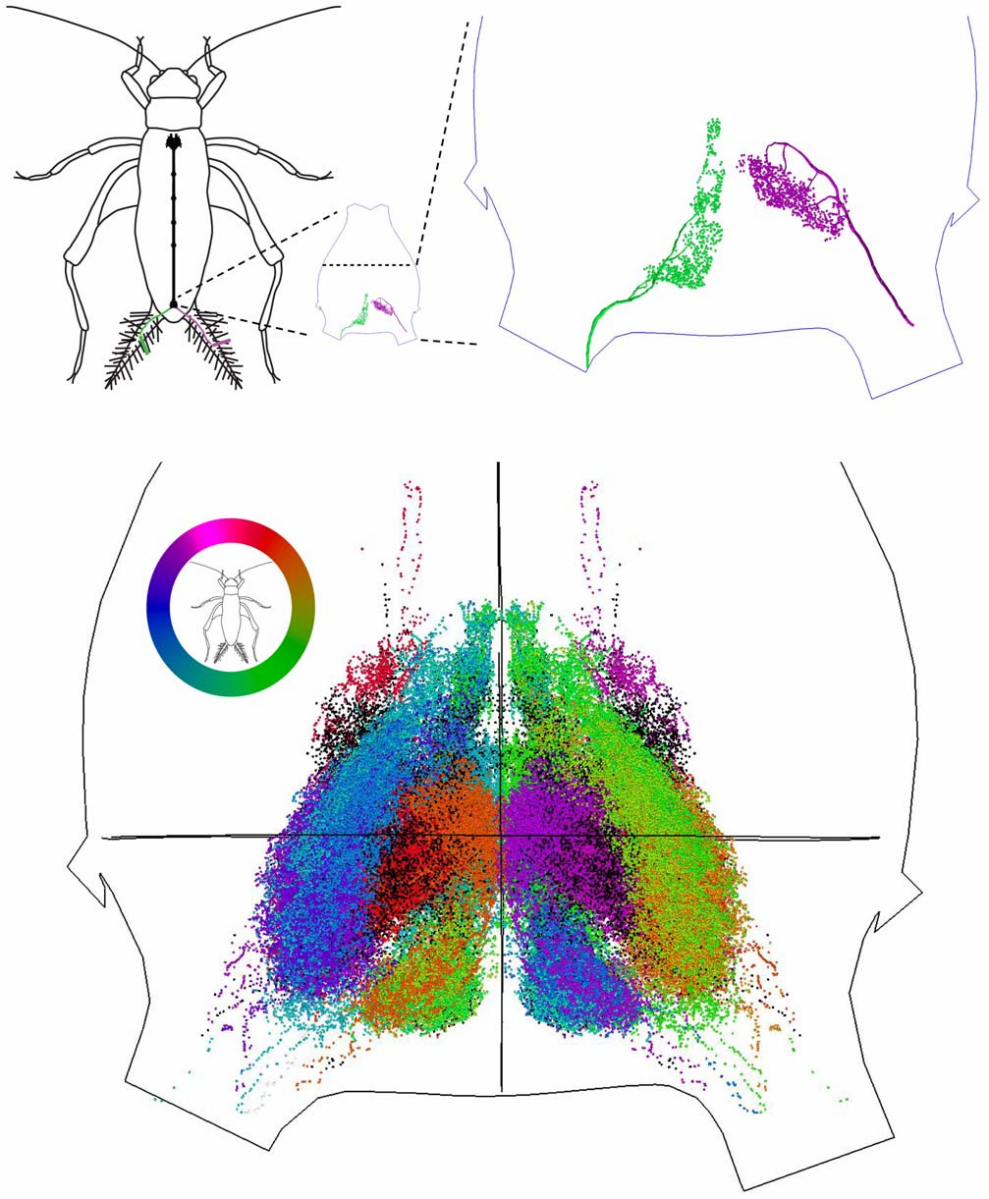
The afferent terminals locus of *Acheta Domestica*. (Upper left) Axons of afferent neurons, which are attached to filiform hairs, project into the terminal ganglion. (Upper middle and upper right) Each axon branches into a tree-like structure in which individual branches are covered by synaptic boutons that provide connections with interneurons in the terminal ganglion. (Bottom) The entire collection of synaptic boutons over all experimentally examined filiform hairs. The color wheel represents the preferred direction of motion of a hair, which correspondingly represents the preferred response direction of the afferent neurons. (Data generously shared by G. Jacobs and collaborators).

The locus of afferent terminals in the terminal ganglion of a cricket is a well-defined object with complex structure. Afferent terminals of hairs that respond to air movement from different directions 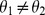 map to different positions. As 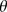 sweeps all angles, this position continuously changes [4]. Each cercus hair is distinguished by its response angle, by its position on the cercus and by its length. Our goal is to describe how the structure of the ganglion is stratified by hair position and hair length. This structure, superimposed on the dendrite structure of the downstream interneurons, constrains the response characteristics of these interneurons [6]. This result uses only the directional response characterization of the hairs and relies on the fact that the afferent terminals corresponding to similarly oriented hairs clearly cluster in the same location (illustrated with the color coding in Figure 2). Is the position of the hair along the cercus and the length of the hair similarly encoded in the structure of the afferent terminals locus? If so, then the interneurons connected to afferents in a particular location will receive input from a class of hairs of a certain length or a certain position on the cercus, and thus this information is available to the interneurons. We note that even if such a structure does not exist, it would still be possible for interneurons to connect preferentially to specific classes of afferents, but the developmental control of the connection process would have to be very complex. A stratification by hair length shows that the structure is more complex, and harder to interpret, than the stratification by response direction. Therefore, more sophisticated methods are required to reveal the stratification structure with respect to the length and position of the hairs.

A casual look at the data set (see Figure S2 for a full three dimensional data set, which can be rotated using an appropriate software) reveals that the defining characteristics of the set are the number and size of the voids, or tunnels, in the point cloud. The data sets (short, medium and long proximal hair terminals; long distal hair terminals), which we will present in this paper, all have a vaguely similar overall shape and structure. However, as we put together different combinations of these data sets, we seem to get varying numbers and sizes of tunnels. How do we quantify the number and size of tunnels in a data set consisting entirely of points? The answer to this question is complicated by the uncertainty in the data, which had been collected from multiple animals and multiple hairs. How do we decide whether the medium and short hair collections map to the same area or not, especially if both the medium and short hairs came from different animals and were themselves all of different lengths? Therefore the need for a robust method with respect to noise is balanced by the need for a sensitive method that is capable of detecting small changes on a local scale, which may have a large effect on a scale of the entire locus. If the terminals of the medium hairs are slightly shifted with respect to terminals of the short hairs, then this small local change may have a large effect on significantly diminishing the size of the tunnel in the terminals of the short data set.

The last challenge stems from the fact that some of the holes and tunnels in the terminal clouds are a result of the functional constraints of the terminal ganglion. Since dendrites of the downstream interneurons must access the terminals of the afferents to make synaptic contacts, some, or probably most, of the tunnels are access points of these dendrites. Therefore, we have to be able to distinguish between the non-essential tunnels, which serve as access points to the afferent terminals, and the essential tunnels, which are a consequence of removing a particular class of terminals.

In view of these constraints and challenges, we identify the essential tunnels in the point cloud data sets as those that correspond to the persistent homology of the data sets. As is explained next, each essential tunnel is in one-to-one correspondence with a circle lying within the data cloud that surrounds the tunnel.

### 1.3. Persistent Homology

In this subsection we outline in lay terms our basic approach to data analysis of a point cloud. We will leave a more formal definition of the concepts of homology and persistence for Section 4.2. For the purpose of an introduction, it is sufficient to note that the homology calculation for each set 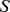 in 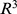 will efficiently compute three groups of embedded objects known as generators. The generators in each of these groups are topologically distinct, which means that one cannot deform any given generator onto any other generator within the set 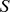. The generators in the first group (the 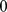
th homology group) represent connected pieces of the set 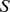. We note that each component of 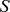 will have one generator of the first kind embedded in it. The generators of the second group (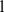
st homology) are circles that circumscribe tunnels in the set 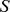. Finally, the generators of the third group (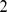
nd homology) are represented by spheres that surround voids in 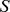. The number of generators in each of these three groups are referred to as *Betti* numbers, and denoted 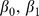 and 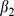, respectively.

**Figure 3:**
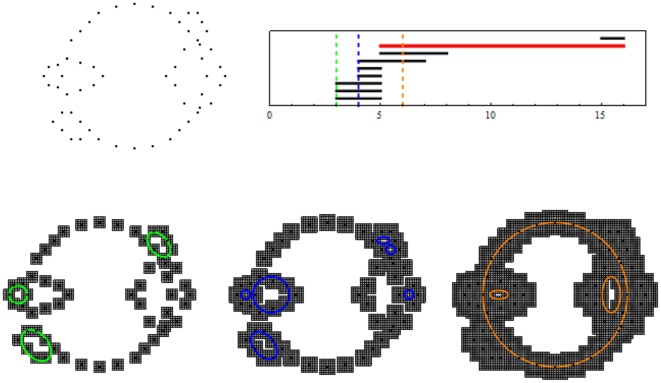
Persistent cubical homology. (Top, left) An example of a point cloud. (Bottom, left to right) As the size of the squares (in arbitrary units) around each point in the point cloud increases from 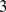 (left), through 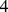 (center) to 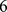 (right), different holes open and close in the gray set of squares. The colored circles around the holes represent generators of the first homology group, which are graphed as a function of the size in the barcode (top, right). The dashed lines are color coded and correspond to the figures in the bottom row. The red line in the barcode represents a *persistent* generator which indicates a hole that is present for a substantial range of sizes of the squares.

It is not a priori clear for how one can apply the concepts of homology to a data set in the form of a point cloud in a nontrivial way. The homology of a point cloud, when considered as a topological space, is entirely straightforward: the number of 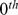 homology generators is equal to the number of points, and there are clearly no higher order generators (circles, or spheres). Imagine, however, that if the points of a point cloud were drawn from a distribution that is centered around a large circle with a standard deviation that is less than, but comparable to, the radius of that circle. Then, can we use the ideas from homology to “discover” this circle from the collection of this noisy data set? The idea of topological persistence [2], [3] elegantly addresses this issue. We introduce a parameter 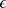 and perform the following construction for a sequence 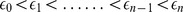 of increasing values of 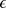. We center a box of the dimension of the ambient space and side 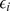 over each data point. We call the union of these boxes a *complex* 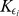 and compute its homology. We note that if 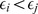 then 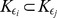. While we refer the reader for detailed definitions of persistent homology and persistent generators to the original literature ([1], [3], see also section 4.2), the main idea is very intuitive and is captured in Figure 3. For a very small 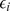 the topology, and hence homology, of the complex 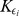 will be identical to that of the underlying point cloud. But as we increase 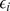, some of the boxes will start to intersect, and as a consequence, 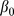 will start to decrease. At some value of 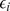, the complex 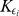 will include a generator of the first homology group that corresponds to the underlying circle of the sample distribution. As 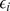 changes, we can track the behavior of generators as a function of 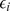. In particular, under very general conditions (see section 4.2) it is possible to pair births and deaths of generators in such a way that each generator born at some value 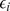 disappears at some larger value 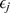. We call the difference 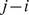 a *lifespan* of that particular generator and 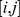 the *persistence interval*. The information about lifespans can be encoded as a *barcode* of a particular point cloud and it can be used to distinguish essential topological features of the cloud from spurious features introduced by experimental and measurement noise. In particular, generators that capture essential topological features will have distinguishably longer lifespans than those that characterize spurious features. These are called *persistent generators*. We will use persistent first homology generators to understand the robust structure of the locus of afferent terminals.

It is worth noting that there is no data-independent threshold that one can use to separate persistent and non-persistent generators. It is only in the context of a given data set that one can define some generators persistent, and this choice is necessarily arbitrary. Our choice led to a conclusion that the combined set of all afferents does not have any persistent homology (i.e. any essential tunnels). This is an attractive conclusion, which is consistent with all our other computations, and which has interesting consequences that we elaborate upon in the discussion. However, this conclusion is ultimately the result of an arbitrary cut-off that separates persistent and non-persistent generators.

## Results

Before presenting our results in terms of persistent first homology generators, we summarize the results in terms of essential tunnels in the point cloud data set. An essential tunnel is encircled by a persistent generator of the first homology group.

Our computations reveal that the afferent terminals of both short and medium hairs have three essential tunnels. However, when we combine the short and medium data sets, the resulting set contains only two essential tunnels. Therefore, the three tunnels in the medium set are not the same as the three tunnels in the short set. This means that at least one tunnel in the short set is filled (or rendered non-essential) by the medium set; and at least one tunnel in the medium set is filled by the short set.

The long proximal set has four essential tunnels; whereas, the combined set of long proximal and long distal afferent terminals contains only two essential tunnels. Therefore, the long distal set fills two tunnels in the long proximal set. Our long distal data set contains substantially less points than the long proximal set. Consequently, it is entirely possible that if we had more data for the long distal set, one or both of the remaining tunnels in the long proximal set would be filled. To address this issue we construct a Gaussian Mixture Model (GMM) for both long proximal and long distal hairs. We then sample these models to create an enhanced set of terminals. The analysis with the sampled data yields the same results as analysis of the experimental data: the combined sampled set contains two essential tunnels.

A combined proximal set, consisting of afferent terminals of all hair lengths (short, medium and long) has only one essential tunnel. This means that the addition of the long proximal class to the combined, short and medium, class fills one essential tunnel.

Finally, the set of all terminals has no essential tunnels remaining. This means that the terminals of the distal hairs, while sparse, fill in the last essential tunnel in the combined set of proximal afferent terminals. We obtain the same result by using either the sparse experimental distal data or substantially larger distal data sampled from a GMM model. As we have noted before, there are still multiple tunnels left in the set of terminals, but these are not essential and likely serve as the interneuron dendrite access points.

### 2.1. Data modeling and filtering

We analyze the database of afferent terminals that is based on the work of Jacobs and collaborators [4], [5], [6]. Recently more data has been added to the database, and some of the data has been refined and corrected. We obtained permission from G. Jacobs and J. P. Miller to use this database.

The total number of data points in the terminal locus is divided into two large categories: terminals of afferents from proximal hairs [4], [5] and, more recently added, terminals of afferents from distal hairs. The proximal hairs are located within the nearest 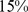 of the length of the cercus to its base, whereas the distal hairs are located beyond the nearest 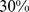 of the length [7]. We will label the corresponding data sets *proximal* and *distal*. The proximal data set is further divided by the length of the contributing hairs into three subclasses, which we will label as long (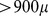
m), medium (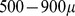
m) and short (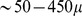
m) [4], [5], [6]. The proximal data set totals to 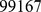 data points divided among the three length subclasses. There are 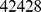 data points representing the terminals of afferents attached to long hairs, 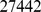 points for those attached to medium hairs and 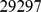 points for those attached to short hairs. In addition to length, each hair responds preferentially to a particular direction of air motion. We split these directions into 13 classes and assign to each data point the class of the corresponding hair.

Distal hairs are all long and are sparser than any other data set. Therefore, we split the preferred angles of the distal hairs into only 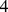 directional classes. Since the taper of the cercus makes access to afferents in the distal part of the cercus difficult, the database only contains 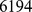 distal hair data points. In order to address the discrepancy between the number of proximal and distal points, we have developed a Gaussian Mixture Model (GMM) that uses one GMM per each of 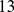 directional classes for long proximal hairs, one GMM per each of 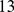 directional classes for medium proximal hairs and one GMM per each of 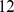 directional classes for small proximal hairs (one class has no data points). We used 4 GMM's for the distal hairs: one per each direction class. This modeling has allowed us to sample additional distal points as to supplement the sparse experimental distal data.

### 2.2. Data filtering

We apply preliminary filtering to the data sets in order to accomplish two goals. The first stems from our main goal of trying to describe topological features of the point clouds given by the afferents of different classes of hairs. These features are defined by the location of the higher density region of the point cloud. Therefore, we aim to reduce the number of outliers that are caused by experimental and/or developmental noise. The presence of such outliers, in what would otherwise be void areas of space, can decrease the lifespans of persistent generators. The second goal of our filtering is to remove redundant points from the dense regions of our point cloud. These points, generally, do not affect the overall shape and topological features of the set; however, their inclusion can significantly slow down the computations.

Our filter 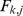 has two parameters 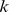 and 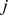. When applied to a point set, it will keep those points that have at least 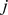 neighboring points within a distance of 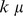
m. This filter is described in more detail in section 4.1. We experimented with many different choices of 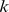 and 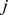. Our goal was to find a combination of 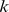 and 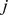, that would delete 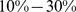 of the least dense points in each of the long proximal, medium proximal and short proximal categories, while keeping a percentage of data points that was similar for each. We chose the filtering function 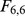, which keeps points having at least 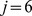 neighboring points within a distance of 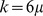
m. This choice led to a reduced long proximal set with 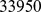 points (which is 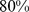 of the original), a medium proximal set with 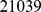 points (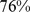) and a short proximal set with 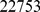 points (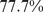). For all data sets, unless otherwise noted, we have applied the 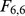. Thus, the long, medium and short data labels, will refer to these filtered data sets for the remainder of this paper.

Our second justification of the use of filter 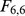 was achieved through a comparison of the resulting persistent generators of the long proximal data set with those of the reduced long proximal data set. This comparison is given in Table 1. The table has the following layout, shared by all subsequent tables in the paper: *data* refers to the input data set, with *length* referring to the length of the lifespan of generators throughout the filtration. The columns, labeled with a number 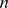, record the number of generators in the filtration of each corresponding data set that had a lifespan of exactly 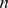. We observe that the proximal long data set has 4 generators, which have distinguishably longer lifespans (three of length 11 and one of length 14). The filtered proximal long data set retains all four persistent generators. Furthermore, the gap in the lifespan length between these four generators and that of any other generator has been widened. This indicates that the filtering works as desired: enhancing the features already present in the data.

**Table 1:** Comparison of filtered and unfiltered data.

Data 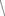 Length	2	3	4	5	6	7	8	9	10	11	12	13	14	15	16	17	18	19	20	21	22	23
UnFiltered Long	78	13	8	4	3	2	2	0	0	3	0	0	1	0	0	0	0	0	0	0	0	0
Filterd Long	15	11	4	3	1	1	0	0	0	1	0	1	0	0	0	0	0	1	0	0	0	1

The first column describes the data set, the top row is the length of the lifespan of generators. An integer entry 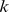 in the column labeled 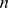 indicates that there is 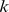 generators with a lifespan of exactly 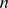.

### 2.3. Topological structure of afferent terminals

We describe the topological structure of the terminals of afferents by computing persistent generators for various subsets. These subsets are formed in four steps.

1. We form reduced (i.e. filtered) sets consisting of long proximal, medium proximal and short proximal hairs separately.
2. We combine the sets of short and medium hairs.
3. We combine together long proximal and (long) distal hairs. However, since the number of data points is much smaller for distal hairs, we will use both direct comparison of the reduced data sets, as well as a comparison using data sampled from a Gaussian Mixture Model.
4. We put together experimental data sets from (2) and (3) to reconstruct the entire afferent terminus.

There are two natural ways to combine any two sets of data. The first approach is to combine the original data sets, then reduce this combined data set using the 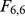 reduction. The second approach is to combine the reduced data sets; that is, we apply the 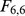 reduction to each individual set, then combine the reduced data sets. While we will not display results for both approaches, we have found that there are minimal differences between the results of either reduction approach. In particular, the number of persistent generators is the same for each approach. Therefore, throughout the rest of the paper we will combine the data sets using the second approach.

#### 2.3.1. Topology of short, medium and long proximal data sets

**Figure 4:**
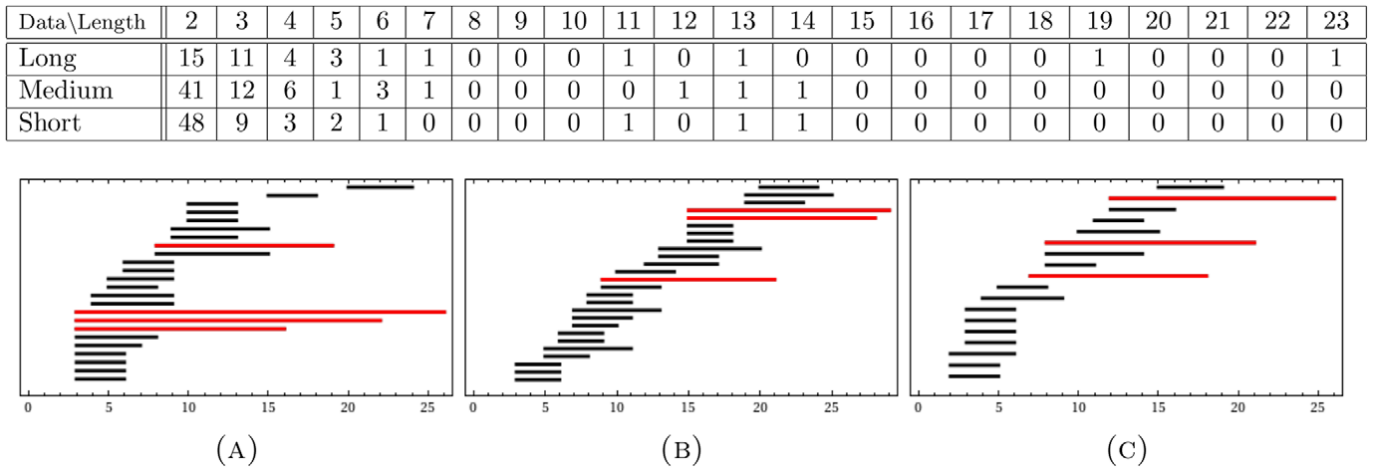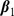-persistence intervals (only lifespans 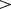
2 are shown) for the reduced proximal (A) long; (B) medium; and (C) short data sets.

We compute persistent generators of the reduced set of long proximal, medium proximal and short proximal hairs. The results are given in the following two forms (Figure 4): A table collecting the number of generators of a given length and a 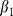-barcode that displays lifespans of each generator. In the 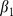-barcode we highlight in red the persistent generators.

We interpret the existence of a persistent generator as evidence for the presence of a significant tunnel-like void in the data. The data presented in Figure 4 shows that

1. The reduced long proximal set has 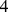 persistent generators with lifespans of 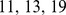 and 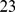.
2. The reduced medium proximal set has 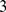 persistent generators with lifespans of 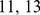 and 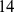.
3. The reduced short proximal set has 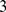 persistent generators with lifespans of 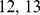 and 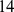.

Therefore, our results show that there are four significant tunnels in the long data set and three, each, in the short and medium data sets.

A natural question is whether the generators for the short and medium data sets are the same, and if, in addition, those generators are a subset of the generators of the long proximal set. To address this question, we combine the medium and short hairs into a single set, and then compute persistence. If the three generators of the short data set are equivalent (homologous) to the three generators of the medium data set, then the number of generators of the combined set will remain three; however, if some of these generators are homologous to zero in the combined set (i.e. the points in one set “fill the hole” in the other set), the combined data set may have a different number of persistent generators.

**Figure 5:**
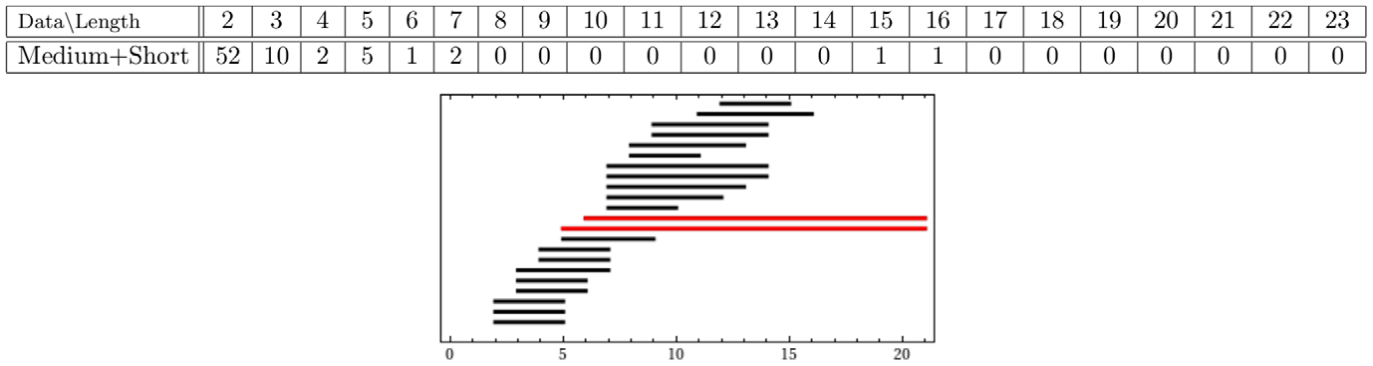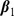-persistence intervals (only lifespans 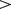
2 are displayed) for the reduced proximal combined medium+short data set.

**Figure 6:**
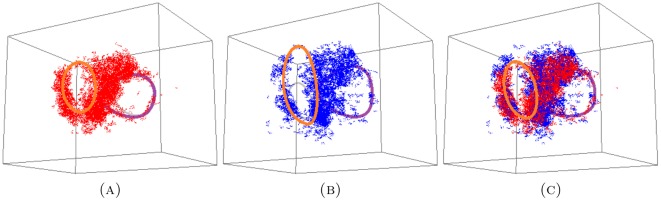
The experimental data for (A) small; (B) medium; and the combined (C) medium+small sets. There are three persistent generators in both the small and medium data sets, but only two persistent generators in the combined set. These two are consistent with two (orange and purple) of the three generators from both the small and medium sets. The voids corresponding to the third generator (grey) in both the small and medium sets are filled in the combined set. In this perspective only the orange persistent generator clearly encircles a void in the data. For a different perspective, showing clearly the purple generator, see Figure S1 and Figure S2.

The persistence results of the combined medium+small data set, as displayed in Figure 5, show that two persistent generators remain after the combination of the two proximal sets. To illustrate what this information means for the data, we display the short, medium and combined point clouds with generators in Figure 6. The combined cloud is displayed in a different orientation in the Figure S1. The respective point clouds of the short and medium data sets seem to occupy approximately the same space, although appear to be slightly offset.

In conclusion, one of the persistent generators for the medium set is filled by the terminals from the short hairs; along with one of the persistent generators of the short data set being filled by the terminals of the medium hairs. This filling appears to be attributed to the two point clouds being slightly offset. The remaining tunnels, characterized by the orange and purple generators in each set, line up enough to allow for the corresponding two generators to remain persistent in the combined point cloud.

#### 2.3.2. Topology of long proximal and long distal sets

Next we look at combining the reduced long proximal data with the distal data. The main issue we need to address is the large discrepancy between the number of points in the long proximal and long distal sets. Since there are more long proximal hairs than long distal hairs, some, but not all, of this difference can be attributed to experimental accessibility. Therefore, in addition to investigating the topology of the union of the two experimental data sets, we will also create a Gaussian Mixture Model (GMM) for both data sets. We then sample a large number of points from each desired data set, process this data set in the same way as the experimental proximal data and compute the persistence for each set separately, as well as for the combined set.

#### 2.3.3. Comparison of experimental data sets of long hairs

**Figure 7:**
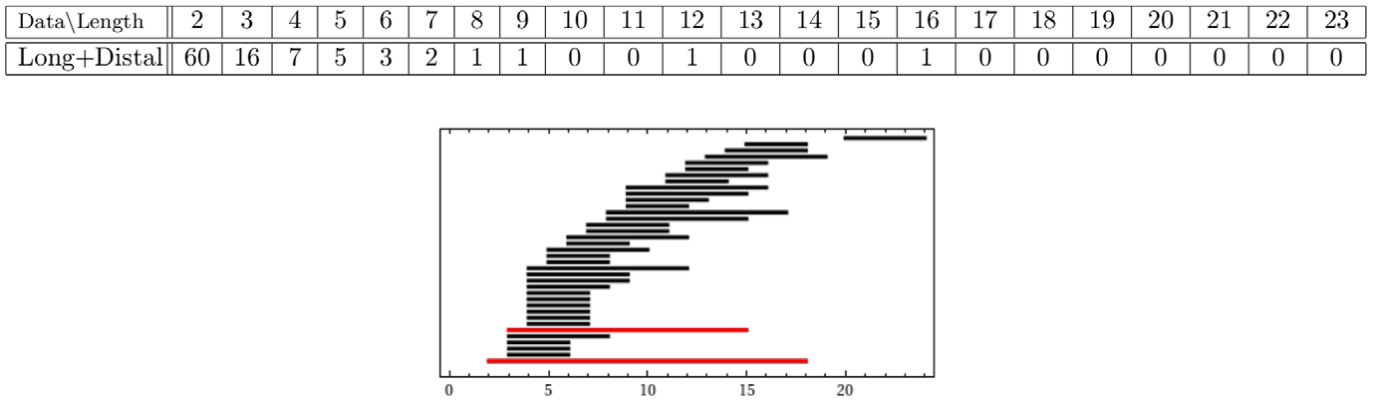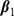-persistence intervals (only lifespans 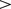
2 are displayed) for the combined long+distal data set.

**Figure 8:**
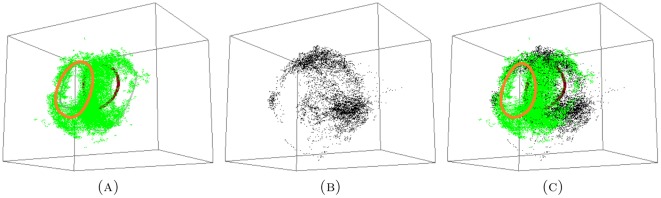
The experimental data for (A) long; (B) distal; and the combined (C) long+distal set. There are four persistent generators for the long set, two of which are consistent between long and long+distal sets (orange and crimson). Grey generators in the long set are not persistent in the combined set. We did not compute the generators for distal set since the data is much sparser than that of the long set.

Since the distal point cloud is made up of only 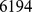 data points, we do not perform any additional reductions on it. Instead, we combine the entire distal point cloud with the data set of the reduced long proximal hairs, then compute the persistence of the resulting set. As we see from the results in Figure 7, the combined point cloud has 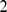 persistent generators with lifespans of length 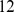 and 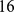. Recall that the proximal long point cloud has 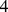 persistent generators: two with longer lifespans (19 and 23) and two with shorter lifespans (11 and 13). The addition of the distal data destroys the two smaller persistent generators, while reducing the lifespans of the other two generators from 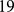 and 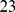 to 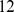 and 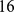. In our interpretation this means that the distal data fills the tunnels in the proximal long data set that support the two shorter persistent generators, and is lining the tunnels in the proximal long data set that contribute to the two larger persistent generators, thereby reducing their lifespan. However, it is also possible that had we had access to more experimental data for the distal hairs, the additional data would fill the voids corresponding to the two remaining persistent generators of the long proximal data set as well. In an attempt to address this question, we construct a GMM for each hair category: distal and proximal long hairs. We sample from each model to obtain a more numerous distal set as well as a sampled proximal long set for which to compare with. The persistence results of the computations using the sampled data are compared to the persistence results of the experimental data sets and displayed in Figure 7 and Figure 8.

#### 2.3.4. Sampling from the GMM model

As mentioned in the introduction, we constructed 42 Gaussian Mixture Models (GMM): one for each of 13 directional categories corresponding to both the medium and long proximal hairs, one for each of the 4 directional categories of the distal hairs, and one for each of the occupied 12 directional categories of the short proximal hairs. Each GMM has an associated weight 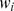, 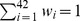, reflecting the relative size of the corresponding class of hairs. Furthermore, each model is itself made up of 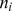 Gaussians with corresponding mean 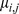 and covariance matrix 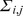. Each Gaussian has an individual weight 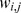 with 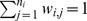. Given an overall desired number of points 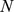 to be sampled, we sample 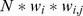 (rounded to the nearest integer) data points from each Gaussian.

#### 2.3.5. GMM for distal hairs

In the last step we sample from the complete GMM to create a GMM distal data set and a GMM long proximal set. These samples are then filtered, removing the densest and the least dense parts. Lastly, we compute persistence on each individual data set as well as on the combined set.

We note that sampling any Gaussian will result in a dense sample set close to the mean. On the other hand, since the support of a Gaussian is the entire space 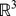, samples will eventually fill arbitrary compact regions around the mean. Therefore, we expect that the oversampling of any particular set would lead to the annihilation of all topological features of the combined long proximal and long distal point-clouds. In order to avoid this oversampling artifact, we combine the initial sampling with two reduction algorithms. The first, designed to eliminate the densest parts of the sampled point cloud, uses *co-density* [1], see section 4.1. The second filter 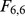, used on all experimental data sets, will eliminate the least dense parts of the point cloud.

We calibrated the co-density filter on the long proximal data set using the results previously displayed for the experimental data and selected the 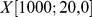 co-density filter (see section 4.1). We will refer to the GMM long proximal data set that was filtered by 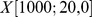 and 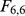 as the GMM long proximal data for the remainder of the paper.

We do not know the appropriate ratio between the physiological number of terminals of the distal afferents and the number of terminals of the proximal afferents of the long hairs. Since distal hairs are sparser, we assume the number of hairs in each of the directional categories is comparable to the minimum population of any proximal directional class. Therefore, we assume that 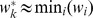 for any class 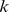 in the four distal classes and 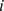 ranging over all proximal directional classes. Due to the uncertainty in the actual numbers, we will create the GMM distal data set by sampling in two different ways.

1. Distal(min) will refer to the GMM distal data set with the weight 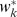 for each distal class chosen to be that of the minimum proximal class weight as described above.
2. Distal(max) will refer to the GMM distal data set with the weight 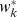 for each distal class chosen at the maximum proximal class weight; that is, 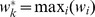, where the 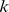
's and 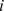
's are as described above.

The first approach leads to a sampling of 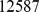 initial data points. The low number of sampled points leads us to use the filtering 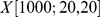, which removes the dense areas as well as the outliers, rather than the filter 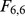 which had been used on all other data sets. Therefore, in removing the most dense 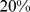 and least dense 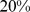 of the sampled points, we obtain the set distal(min), which contains 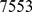 data points.

The second approach generates 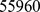 initial data points. After removing the densest 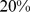 of the points using the 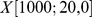 filter, we arrive at 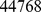 data points. With subsequent filtering by the 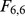 reduction, we obtain the data set distal(max), which has a cardinality of 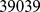 points (when 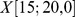 was used in the first step the set contained 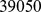 points). The results of the computation of persistence for these sets, as well as each's combination with the GMM proximal long set, are in Table 2.

**Table 2:** Persistent Generators for GMM Data Sets.

Data 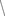 Length	2	3	4	5	6	7	8	9	10	11	12	13	14	15	16	17	18	19	20	21	22	23	24	25	26	27
Distal(max)	20	8	2	2	0	2	0	0	1	0	0	0	0	0	0	0	0	1	0	0	0	0	0	0	0	0
Distal(min)	19	4	3	3	0	0	0	0	0	0	1	0	0	0	0	0	0	0	0	0	0	0	0	0	0	0
Long	19	7	4	2	2	1	0	0	0	1	0	1	0	0	0	0	0	0	0	0	0	1	0	0	0	1
Long+Distal(max)	35	16	2	1	3	0	0	0	0	0	0	0	0	0	0	1	0	0	0	1	0	0	0	0	0	0
Long+Distal(min)	40	9	7	1	2	0	1	0	0	0	0	0	0	0	0	1	0	0	0	0	0	0	1	0	0	0

Number of generators with prescribed length for sampled distal hairs(at different weights), sampled long proximal hairs and the combined sampled long proximal and distal sets.

Observe that distal(max) has two persistent generators with lifespans of 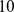 and 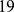; whereas the Distal(min) data set has a single persistent generator with lifespan 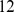. However, when we add these sets to the GMM long proximal data set, the resulting combinations both yield 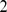 persistent generators with lifespans of 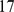 and 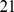 for the combination using distal(max) (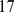 and 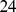 when using distal(min)). These are the same results as we obtained for the combined experimental data sets in Figure 7 and Figure 8.

We conclude that

**Figure 9:**
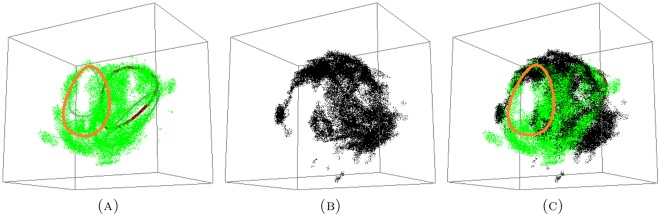
The GMM data for (A) long proximal; (B) distal(max); and (C) the combined long+distal(max) set. There are four persistent generators for the GMM long proximal set, two of which are consistent between the GMM long and GMM long+distal sets (orange and crimson). Grey generators in the GMM long proximal set are not persistent in the combined set.

- The experimental long proximal set and GMM long proximal set each have 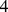 persistent generators, with lifespans of 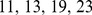 and 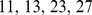, respectively.
- The addition of either the small sample (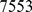 points) or large sample (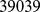 points) of the GMM distal cloud to the GMM long proximal data fills the voids corresponding to the two smaller persistent generators, while reducing the lifespans of the other 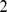 larger persistent generators. Therefore, we obtain the same result as with the experimental distal data: the (GMM) distal data is “lining” the tunnels of the (GMM) long proximal set, corresponding to the 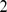 more significantly distinguished persistent generators, see Figure 9.

### 2.4. Combined proximal set

**Figure 10:**
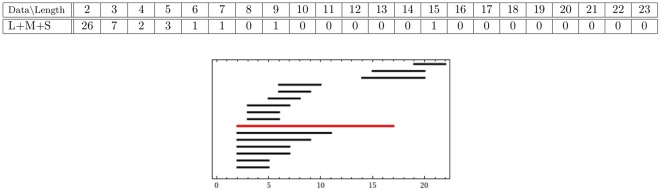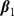-persistence intervals (only lifespans 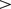
2 are shown) for the reduced experimental proximal combined long+medium+short data set.

We create the proximal data cloud that includes afferent terminals from proximal hairs of all lengths and all orientations by combining the experimental data for proximal long, medium and short hairs. The results of this combined experimental proximal data cloud are in Figure 10.

We note that there is only one persistent generator. Therefore the addition of the long proximal data set to the combined short and medium data set has annihilated one of the persistent generators in that latter set and three of the persistent generators in the former set.

### 2.5. Entire data set

Finally, we compute persistent generators for the entire set of terminals. In doing so, we analyze three data sets. The first is the complete experimental data set that combines the combined data sets of long+distal and medium+small. Then, because the experimental distal point cloud is so sparse, we also replace the experimental distal data with the two sampled GMM distal data sets. Recall, the sampled data sets are filtered in the way described in section 2.3.5. We add the distal(min) and distal(max) data sets to the experimental long+medium+short data set to form the combined(min) and combined(max) data sets, respectively.

**Figure 11:**
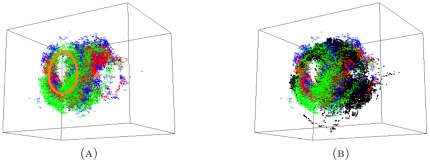
The experimental data for (A) long+medium+short; and (B) long+medium+short+distal. The combined set of all proximal hair afferent terminals has only one persistent generator ((A), orange), while the entire set does not have a persistent generator.

**Figure 12:**
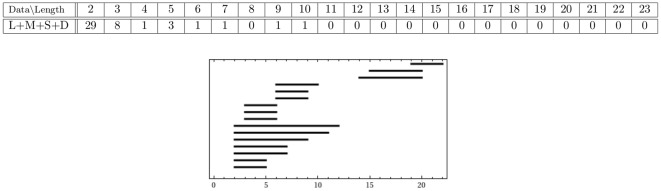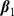-persistence intervals (only lifespans 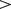
2 are shown) for the combined experimental set.

The persistent homology results for the experimental combined set are displayed in Figure 11 and Figure 12. There are no longer any persistent generators. Recall that the combined proximal long+medium+short set does have a single persistent generator. Therefore, the computations reveal that the sparse experimental distal afferent terminals make the distinguishable tunnel in the proximal data smaller. A close inspection of (B) of Figure 11 shows multiple terminals within the central tunnel (black dots) in the data cloud. These are distal terminals within the void in the proximal set. This observation puts in question the robustness of our method and result. Recall we did not filter the outliers from the experimental distal set. To that end, if these few terminals are the main cause of the loss of persistence of the proximal generator (orange in (A) of Figure 11), our result, and method, could not be considered robust. To address this issue, we analyze the combined(max) and combined(min) point cloud data sets.

**Figure 13:**
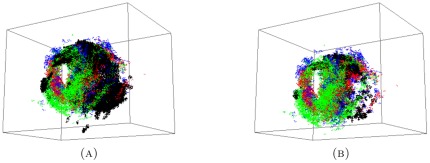
The data sets for (A) Combined(max); and (B) Combined(min). There are no persistent generators in either combined data set.

**Figure 14:**
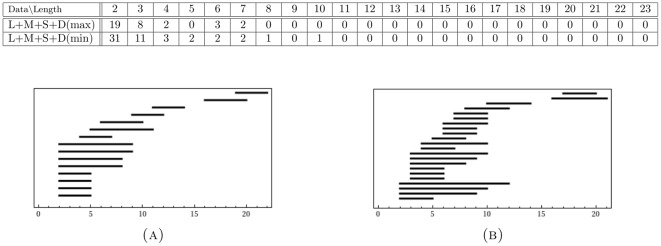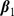-persistence intervals (only lifespans 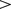
2 are shown) for the combined data sets: (A) Combined(max) and (B) Combined(min).

The results of these persistence computations are displayed in Figure 13 and Figure 14. The comparison between combined(min) and the experimental combined set (Figure 12) reveals almost an exact match. The more interesting comparison is between combined(max), which may represent the true physiological abundance of the distal terminals, and the experimental combined set. We note that the lifespans of all the generators are shorter for the combined(max) set than are those for the experimental combined set, in spite of the fact that the there are no longer any visible distal terminals within the central tunnel. The terminals visible in the tunnel in Figure 11 were filtered out as outliers. This shows that these points were not the main cause of the loss of persistence of the proximal generator (orange in (A) of Figure 11). Rather, the distal terminals are lining the inside walls of this tunnel, and thus, reducing its size. This result confirms that the loss of persistence of the proximal generator from the addition of the distal data points is a robust phenomena.

We want to emphasize that there are still multiple homology generators in the combined data sets (see Figure 12 and Figure 14) that correspond to different tunnels in the point cloud; however, there is no persistent generator that is robustly present in the data. These remaining tunnels very likely contain the dendrites of the interneurons in the terminal ganglion, which are downstream from the afferents, and which make synaptic contact with the afferent terminal cloud.

## Discussion

The lack of persistent generators in the combined proximal and distal set illustrates that the set of terminals of all hairs has no significant tunnels or voids. This result is not surprising from an efficiency standpoint: the space in the terminal ganglion is limited and the terminals of afferent neurons are filling the entire available space. The potential consequences for neural processing are more intriguing. Given that the directionally sensitive interneurons (IN) from the ganglion are sampling the terminals of afferents with the same directional sensitivity, and since it is likely that the three dimensional position of both dendrites of IN's and afferent terminals are developmentally determined only up to some finite precision, the spatial separation of afferent terminals puts the limit on the precision with which the downstream processes can distinguish direction of the air motion. Since the angles form a circle (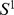), the afferent terminals must be embedded in the ganglion as an approximate image of a full torus 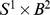. Here 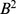 is a disc that approximates the spatial extend of the afferent terminals with the same response angle. The need of separating the terminals of hairs with the nearby response angle to enhance acuity of the system, is balanced by the need to pack the terminals in the smallest possible volume: a ball 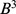. These two needs are not compatible. There is no embedding of the full torus to a 3-disc 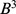; the space 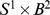 has a different homology than the space 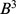. As a consequence, there must be at least one point in 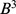 where the directional tuning is not well defined. This is similar to topological singularities (pinwheels, vortices) in a primate cortical striate cortex, which result from the fact that 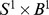 cannot be embedded into a two dimensional disc 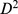. The only difference is that the pinwheel in the terminal ganglion is three dimensional, while spatial orientations of columns in a striate cortex is a two dimensional phenomenon.

It is interesting to note that the distal hairs are filling the last essential tunnel in the combined proximal set. Since distal hairs are more sparse, not all directions are represented in the set of their preferred directional responses. One can speculate that the hairs further along the cercus fill more and more central positions along the tunnel in the proximal set. If, in addition, there was a hair at the very tip of the cercus, this would be the hair that would map into the pinwheel position in the terminal ganglion. This hypothetical arrangement of terminals (more distal hairs have afferent terminals closer to the pinwheel) would also make sense from another perspective. Our collaborators have recently shown [8] that the cercus acts as a delay line; a signal from greater distal hairs travels to the ganglion significantly (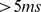) longer than the signal from the proximal hairs. If the system works as a delay line, it makes sense to separate the terminals of the distal hairs from those of the proximal hairs, but still align the directional sensitivities. Our current data does not have the spatial resolution to confirm this conjecture, but our analysis, which shows that the distal hairs line the tunnel in the proximal cloud, is compatible with this theory.

We also comment on our results about the proximal hair terminals. We have shown that while short and medium hairs have three relatively weak persistent generators each, the combined medium-short set has two robust persistent generators. This suggests that our separation of the medium-short set into two groups is artificial and only the combined set has spatial structure that suggests functional relevance. The addition of the long proximal hairs to the medium-short set annihilates one of these generators. The resulting set of terminals has only one generator that encircles the 3-D pinwheel where the angular response is not well defined.

Our goal in this paper was to show the applicability of sophisticated approaches from computational homology to the analysis of noisy neuroscience data. The source of noise is both developmental, encompassing animal to animal variability, and experimental. Our methods provide robust results that give fresh insight into the structure, as well as suggest functional relevance, of the spatial organization of the terminals of afferent neurons.

## Methods

### 4.1. Data filtering

Density estimation is a highly developed area within statistics [9] and, following the lead of Lee *et. al.* [10] (see also the review by Carlsson [1]), we will employ a *codensity function* as well as an outlier-reduction function that we call the *DN-density function*.

The *codensity function* is defined as follows: For any fixed positive integer 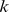 and the point cloud , we define the *k-codensity function* 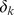 for 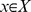 by
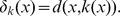
where 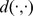 denotes the distance function in 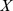, and 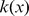 denotes the 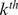 nearest neighbor of 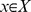. The function 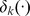 is inversely related with density, since a dense region will have smaller distances to the 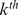 nearest neighbor. Considering that we are interested in dense regions, we will study subcollections of points for which 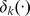 is bounded from above and/or below by given threshold percentages. We also note that each 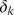 yields a different density estimator, since for large values of 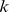, 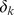 computes density using points in large neighborhoods of 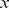; whereas, for small values of 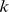, small neighborhoods are used. Therefore, for large 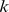, 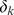 corresponds to a smoothed out notion of density and for small 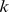, 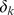 corresponds to a version that carries more of the detailed structure of the data set. Following similar notation as was used by Carlsson [1], we denote a subset 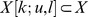, where 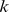 is a positive integer and 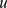 (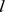) is the upper (lower) percentage bound on the points to keep. More precisely,
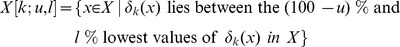
This approach to data reduction will be used exclusively for the data sampled from the Gaussian Mixture Model to achieve a reduction in the densest part of the sampled point cloud. The *DN-density function*, described below, will be used for both the experimental and sampled data in order to eliminate outliers in the least dense regions.

The *DN-density function* is defined as follows: For any fixed positive integer 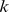 and the point cloud 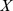, we define the *k-DN density* function 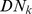 of 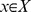 by
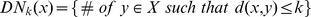
where 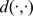 denotes the distance function on 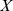 and the integer 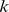 is a distance threshold to the neighbors of 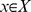. Then, for a fixed positive integer threshold 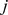, we define the *thresholding* function 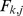 on a set 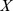 by
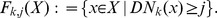
Finally, we let
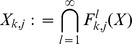
be the intersection of the iterates of the data reducing function 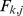. Note that these sets are finite and nested, 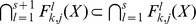, so that the procedure terminates at a finite iteration. In addition, note that for poorly chosen values of 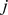 and/or 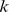, the set 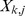 can be empty; for instance, if we select 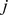 or 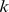 to be extremely large. In practice, the sets stabilize after a few iterations.

#### 4.1.1. Calibration of the GMM filtering

We calibrate our method on the long proximal data set using the results previously displayed for the experimental data. The GMM long proximal data set is created by sampling a total of 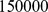 points from the complete GMM as formerly described. We eliminate 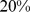 of the points from the most dense regions of the sampled set by applying either the filter 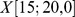 or the filter 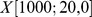. These two different filtering mechanisms, which use significantly different sized neighborhoods, allow us to compare the effect of the smoothness of the co-density function on the selection. The final filtering of the GMM long proximal data set is the same as that of the experimental long proximal data set; that is, we filter the GMM long proximal set using the 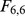 reduction.

Following this procedure, the GMM long proximal data set equates to 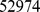 points of the total 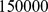. Removing the most dense 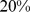, using either of the 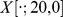 filters, leaves a reduced GMM long proximal data set with 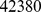 data points. The cardinality of the set compares favorably to that of the non-reduced experimental long proximal data set, which contains 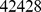 points. The application of the 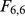 filter further reduces the GMM long proximal data set to a set of 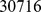 points, if the density filter 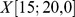 was previously applied, or 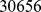 points, if the filter  had been applied. The cardinality of each of these reduced GMM sets compares favorably to the reduced experimental proximal long data set, which contains 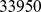 points. The final step of the calibration is to compute cubical persistence on each reduced GMM data set, with results displayed in Table 3, and compare these results to those of the experimental long proximal data as displayed in Figure 4.

**Table 3:** Comparison of GMM long proximal models with different filters.

Data 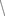 Length	2	3	4	5	6	7	8	9	10	11	12	13	14	15	16	17	18	19	20	21	22	23	24	25	26	27
Long-1000	19	7	4	2	2	1	0	0	0	1	0	1	0	0	0	0	0	0	0	0	0	1	0	0	0	1
Long-15	12	4	2	1	1	1	0	0	0	1	0	1	0	0	0	0	0	0	0	0	0	1	0	0	0	1

Long-1000 refers to GMM sampled long proximal data with filter 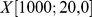.

Long-15 refers to GMM sampled long proximal data with filter 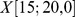.

We observe that the data sets created by both the 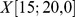 and 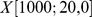 density filters yield the same results: in either case, the GMM long proximal data has 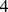 persistent generators at lifespans of 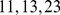 and 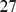. Recall that the experimental long proximal data had 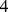 persistent generators at lifespans of 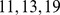 and 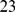. Thus, seeing that we have an equal number of persistent generators for the GMM and experimental data and, in addition, the lifespan length for each is nearly identical, we conclude that the Gaussian Mixture Model is a good model for the proximal long data.

### 4.2. Persistence theory

In this subsection we outline the main concepts of topological persistence, which combines the idea of homology with that of filtration. The homology groups and Betti numbers associated with these groups are computable invariants that have been developed over the last century (see [11] for a relatively accessible introduction). Homology groups form a graded abelian group; that is, there is one such group for every non-negative integer, although for reasonable topological spaces such as compact manifolds, these groups are trivial for integers larger than some finite 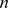. For our purposes it is sufficient to know that the dimension of the 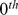 homology group (and thus the 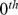 Betti number: 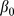) corresponds to the number of connected components of the set, while the dimension of the 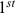 homology group (which in our case will be always equal to the 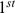 Betti number: 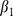) measures the number of one-dimensional holes in the set. A one-dimensional hole is characterized, roughly speaking, by a loop that can be embedded into the set around a hole for which the loop cannot be smoothly (i.e. without tearing it apart) shrunk to a point within the set. We will call such a loop a *generator* of the first homology group.

We now define more precisely the idea of topological persistence in the context of cubical complexes used in this paper. For more details the reader is referred to [2], [3]. Recall that for a sequence 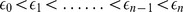 of increasing values of 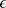, we center a box with a side of length 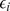 over each data point. We call the union of these boxes a *complex* 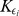 and note that if 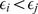 then 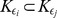. These complexes form a filtration of the complex 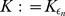. A *filtration* of a complex 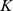 is a nested sequence of subcomplexes that starts with an empty complex and ends with the complete complex 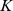


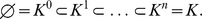
Define 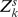 and 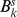 to be the 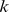-th cycle and 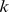-th boundary group, respectively, of the 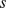 complex 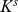 in the filtration. To capture persistent cycles in 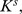 we factor its 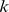-th cycle group by the 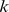-th boundary group of 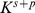, where 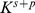 is 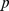 complexes further along in the filtration. Therefore, the 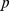 *-th persistent* 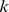 *-th homology group* of 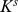 is
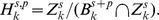
This is well defined since both 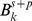 and 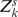 are subgroups of the chain complex 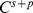 of 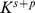, and thus, a group. The 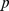 *-th persistent* 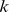 *-th Betti number* 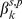 of 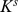 is the rank of 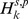.

One can also define the 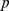-th persistence group using inclusion induced injective homomorphisms of ordinary homology groups. The main observation is that if two cycles are homologous in 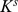 then they still exist and are homologous in 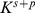. Therefore an inclusion induced map
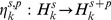
is well-defined and maps a homology class into one that contains it. The image of this homomorphism is isomorphic to the 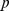-th persistent homology group of 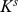,
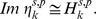



### 4.3. Data analysis

In this subsection, we present the preprocessing and processing used in our computation of persistent homology on each data set. The overall size of each individual data set, the computational memory needed to store the filtration of each data set and the memory needed for the persistence computations has forced us to use cubical homology for our computations (see [12] for relatively accessible introduction). Cubical persistent homology allows for the filtration 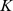 to be created using non-overlapping 3-dimensional basis cubes, which then can be stored as a bitmap, greatly reducing the computational cost [13], [14]. The construction is based on a cubical grid of the part of the space that contains all the data points of the point cloud. In this way, the 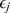
's that parameterize the filtration will all be multiples of the size of the elementary grid element (i.e. *elementary cube* [12]).

We first translate the data into the positive quadrant of 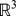. The critical input for this process is the size 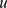 of the basis cube (in 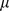
m). An appropriate choice is important for two reasons: if one chooses a basis cube too large, the growing of the filtration is too fast, resulting in a lack of persistent generators; if the cube is too small, the size of the filtration is too large and computationally costly, not providing any further information beyond that obtained from a filtration with a larger basis cube size. The appropriate choice for the vast majority of data throughout this paper was 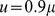
m. However, we had to choose 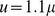
m for the largest of our data sets (those that combined data from all hairs) due to the large computational cost that we incurred at input sizes of 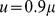
m and 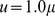
m. Given the entire translated data set, we define 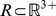 to be the smallest box
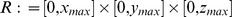
that contains the point cloud data set, for which 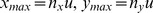, and 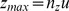 are integer multiples of the basic length 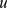. We divide 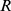 into 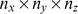 elementary cubes. Each cube
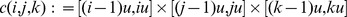
is uniquely determined by its coordinates 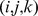.

Next, we build our filtration 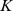. The initial complex 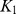 consists of all elementary cubes in 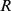 that contain at least one point from the data set 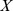. The next step is to increase the size of the elementary box and construct a complex from all larger boxes that contain at least one point from 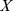. A naive way to increase the size of the elementary box is to simply double its size. The resulting exponential growth of the elementary cubes would yield only a few complexes K in the filtration. An alternative way, which we describe next, creates nested complexes and results in a higher resolution persistence computation. We associate to each elementary cube a positive integer value 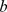, which will be referred to as a *birth time*. Initially, we assign a birth time of zero, 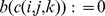, to all cubes. We will then inductively define a nonzero birth time value of 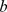 as we construct our filtration.

We start by assigning the birth time value 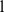 to all cubes that contain at least one point of the set 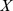


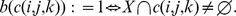
In the inductive step, for each elementary cube with 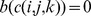, we record all bordering elementary cubes' birth times in the set 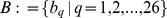. Then, if 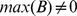, we increment the birth time 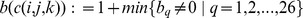. However, if 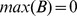, then 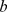 remains unchanged. This process continues until every elementary cube in 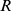 has a nonzero birth time assigned to it. There is a finite number of cubes and, since we only change to a non-zero value of 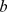 once, this procedure is finite. Let 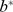 be the maximum birth time across all cubes in 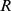. We define the complexes 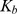, 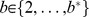 of our filtration 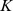 by
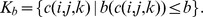
We compute cubical persistence of the filtration complex 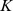 using code developed by Mrozek, Batko and Wanner [13], [14], [15] called *cubPersistenceMD*. While the final computation of homology is based on the classic Smith Normal Form algorithm, the significant computational improvements are found in its preprocessing co-reduction algorithm ([13], [14], also, See Section 4.4). This preprocessing algorithm reduces the overall size of the input data to the Smith Normal Form while preserving the homology of the complex. The (co-)reduction runs in linear time, whereas the Smith diagonalization algorithm has a complexity of 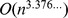 [13], [14], [16].

**Figure 15:**
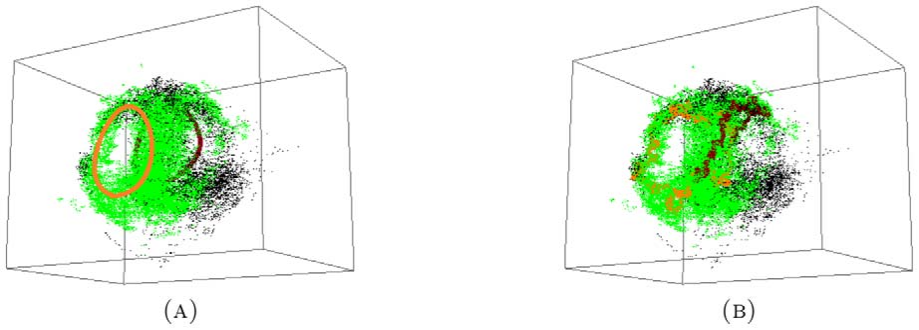
The persistant generators of the experimental data set long+distal are displayed. (A) long+distal with smooth represenative generators; and (B) long+distal with actual generators produced in computation.

In the computations described above, the output of the process is a set of lists of birth/death times of generators in the filtration 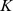. The 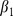-Persistence Intervals, as displayed throughout the paper, are constructed as a result. These barcodes provide detail as to what is happening in the point cloud data set, but in these situations it is often the “where” that is just as important as the “what”. Therefore, we have employed subprograms from the package CHomP [12], specifically *Homcubes*, to aid in acquiring a means to visualize the persistent generators of each data set. The input of *Homcubes* is the complex 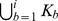. The choice of 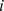 is based on the birth/death times of the persistent generators of each specific point cloud data set; the only requirement is that birth time 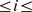 death time. The resulting set of generators is constructed using basis cubes from the complex 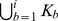, many of which are not present in the initial complex 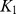. However, we display these generators with the cubes from complex 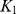, since these cubes closely approximate the actual position of afferent terminals. This often results in generators that seemingly protrude into free space, which is just a consequence of a mismatch between the displayed set 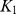 and the set 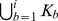 where generators are computed. Generators are also very rarely smooth. Therefore, throughout the paper, we have displayed figures with smooth circular representations of the computed generators. We illustrate the difference in the Figure 15.

### 4.4. Co-reduction algorithm

Given a simplicial (or cubical) complex 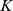 and a free chain complex 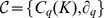 with basis 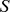, we say

1. A pair 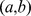 of elements of 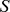 is said to be an ***elementary reduction pair*** if
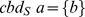

2. A pair 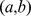 of elements of 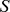 is said to be an ***elementary coreduction pair*** if
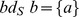

**Theorem** [13], [14], [15]: If 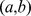 is an elementary reduction or coreduction pair in 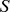, then
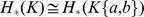



**Function Coreduction (Homology Complex** 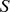 **, a vertex** 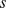 **)** [13], [14], [15]

begin


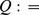
 empty queue of generators;

enqueue(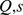)

while 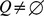 do begin


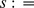
 dequeue(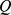);

if 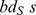 contains exactly one element 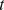 then begin
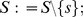



for each 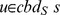 do

if 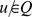 then enqueue(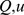);
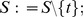



end

else if 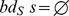 then

for each 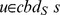 do

if 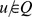 then enqueue(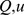);

end;

return 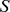;

When implemented as a bitmap, for a cubical complex 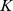 the coreduction algorithm runs in 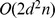, where 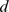 denotes the embedding dimension of the cubical set [13], [14], [15].

## Supporting Information

Figure S1 The experimental data set medium+small is displayed in two perspectives. (A) medium+small data in the perspective that was displayed throughout the paper; (B) medium+small data in a second perspective providing a clear view of the second (purple) persistent generator.
(TIF)

Figure S2
**Each figure is a Mathematica** 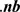 **file containing a 3-D figure.** These figures depict the medium+small point cloud; the medium+small point cloud with the computed generators; and the medium+small point cloud with computed generators and containing the black marked-double-circle, whose arclength can is parameterized by the response angle of the afferent hairs.(NB)
