# Supplementary figures and images for: Structure of the Afferent Terminals in Terminal Ganglion of a Cricket and Persistent Homology
Source: PLoS One. 2012 May 23;7(5):e37278. doi: 10.1371/journal.pone.0037278 (PMC3359360; doi:10.1371/journal.pone.0037278)

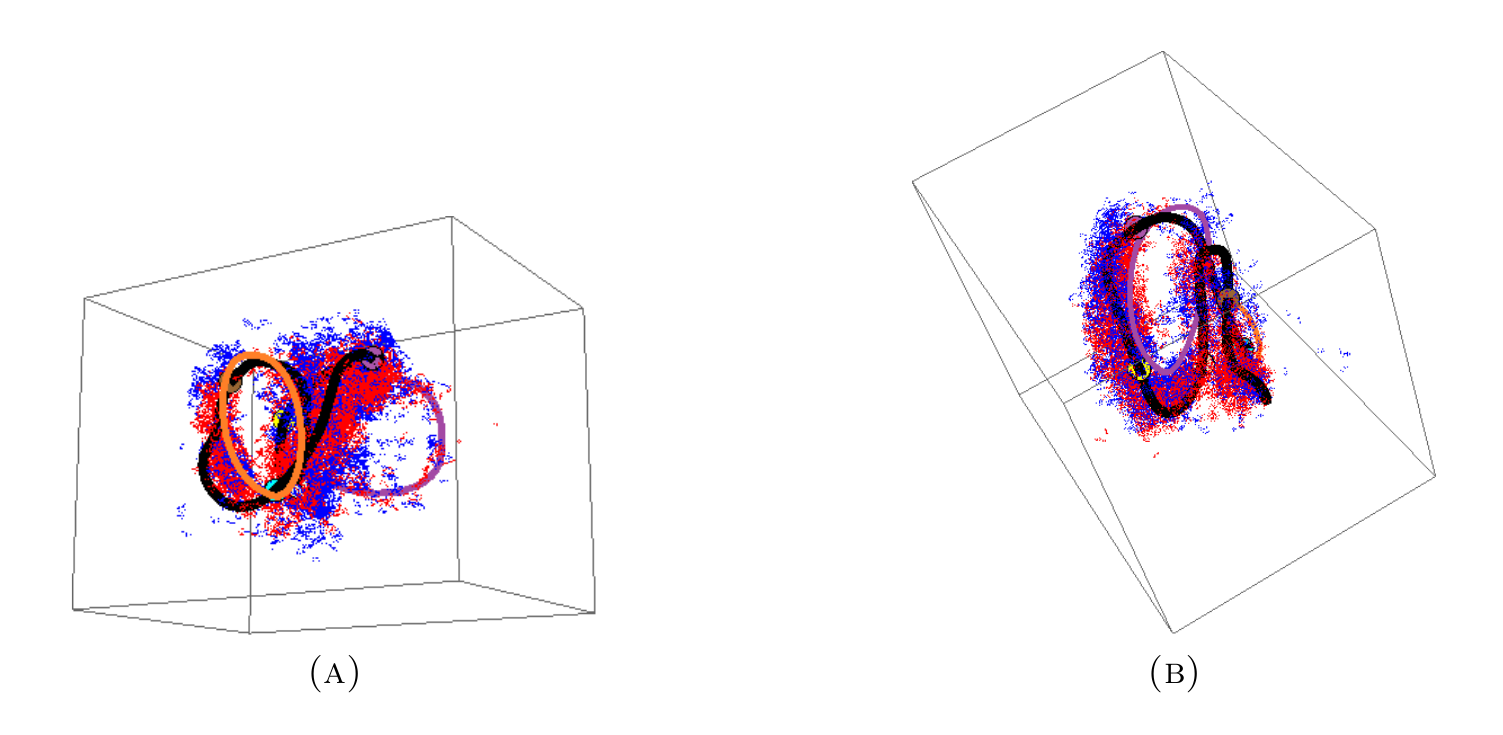

Supplement: Figure S1 — The experimental data set medium+small is displayed in two perspectives. (A) medium+small data in the perspective that was displayed throughout the paper; (B) medium+small data in a second perspective providing a clear view of the second (purple) persistent generator. (TIF) [file pone.0037278.s001.tif]
